# Protease-activated receptor 2 induces ROS-mediated inflammation through Akt-mediated NF-κB and FoxO6 modulation during skin photoaging

**DOI:** 10.1016/j.redox.2021.102022

**Published:** 2021-05-26

**Authors:** EunJin Bang, Dae Hyun Kim, Hae Young Chung

**Affiliations:** Department of Pharmacy, College of Pharmacy, Pusan National University, Gumjung-gu, Busan, 46241, South Korea

**Keywords:** Protease-activated receptor 2, NF-κB, FoxO6, ROS, Inflammation, Skin photoaging

## Abstract

Long-term exposure to ultraviolet irradiation to skin leads to deleterious intracellular effects, including reactive oxygen species (ROS) production and inflammatory responses, causing accelerated skin aging. Previous studies have demonstrated that increased expression and activation of protease-activated receptor 2 (PAR2) and Akt is observed in keratinocyte proliferation, suggesting their potential regulatory role in skin photoaging. However, the specific underlying molecular mechanism of PAR2 and the Akt/NF-κB/FoxO6-mediated signaling pathway is not clearly defined. In this study, we first used the UVB-irradiated photoaged skin of hairless mice and observed an increase in PAR2 and Gαq expression and PI3-kinase/Akt, NF-κB, and suppressed FoxO6. Consequently, increased levels of proinflammatory cytokines and decreased levels of antioxidant MnSOD was observed. Next, to investigate PAR2-specific roles in inflammation and oxidative stress, we used photoaged hairless mice topically applied with PAR2 antagonist GB83 and photoaged PAR2 knockout mice. PAR2 inhibition and deletion significantly suppressed inflammatory and oxidative stress levels, which were associated with decreased IL-6 and IL-1β levels and increased MnSOD levels, respectively. Furthermore, NF-κB phosphorylation and decreased FoxO6 was reduced by PAR2 inhibition and deletion *in vivo*. To confirm the *in vivo* results, we conducted PAR2 knockdown and overexpression in UVB-irradiated HaCaT cells. In PAR2 knockdown cells by si-PAR2 treatment, it suppressed Akt/NF-κB and increased FoxO6, whereas PAR2 overexpression reversed these effects and subsequently modulated proinflammatory target genes. Collectively, our data define that PAR2 induces oxidative stress and inflammation through Akt-mediated phosphorylation of NF-κB (Ser536) and FoxO6 (Ser184), which could be a critical upstream regulatory mechanism in ROS-mediated inflammatory response.

## Abbreviations

Akt: Protein kinase B
COX-2: Cyclooxygenase-2
DAG: Diacylglycerol
DCFDA: 2', 7'-dichlorofluorescin diacetate
FoxO6: Forkhead box O 6
GPCR: G protein-coupled receptor
IL-1β: Interleukin 1 beta
IL-6: Interleukin 6
IP_3_: Inositol 1,4,5-triphosphate
KO: Knockout
MITF: Microphthalmia-associated transcription factor
MnSOD: Manganese superoxide dismutase
NF-κB: Nuclear factor kappa-light-chain enhancer of activated B cells
PAR2: Protease-activated receptor 2
PIP_2_: Phosphatidylinositol 4,5-biphosphate
PKC: Protein kinase C
PLC: Phospholipase C
ROS: Reactive oxygen species
UVB: Ultraviolet B
WT: Wild type

## 1 Introduction

The skin is the largest and most complex organ in the body, which is in direct contact with the external environment. Cumulative exposure to ultraviolet (UV) irradiation damages the skin, leading to photoaging [1,2]. Premature skin aging is characterized by epidermal thickening, hyperpigmentation, coarse wrinkles, angiogenesis, immune and inflammatory responses, and reactive oxygen species (ROS) production [1]. At the molecular level, it is accepted that NF-κB is one of the core transcription factors that becomes activated and plays a critical role in the induction of proinflammatory cytokines, such as IL-6, IL-1β, IL-1α, and cyclooxygenase-2 (COX-2) [3]. During this elevated inflammatory response, intracellular ROS levels increase and ubiquitous targeting can induce oxidative stress even in adjacent cells, leading to molecular oxidative damage [4]. However, the detailed signaling pathways, action mechanisms, and regulatory signaling molecules of this process are not fully defined.

Protease-activated receptors (PARs) are a subfamily of G protein-coupled receptors (GPCRs), and are seven-transmembrane domain receptors comprising PAR1, 2, 3, and 4. PAR2 is cleaved and activated by serine proteases, including coagulation factors VIIa, tissue factor (TF), trypsin, kallikrein, and others. These enzymes cleave the extracellular N-terminus, unmasking endogenous tethered peptide sequences for the receptor binding loop for receptor activation [5,6]. PAR2 is well expressed in the skin epidermis, and receptor activation becomes prominent in UV-irradiated skin and cultured keratinocytes [7]. PAR2 is known to exert regulatory functions in the epidermal barrier, keratinocyte differentiation, cutaneous tumorigenesis, inflammation, and pigmentation [8].Canonical PAR2 signaling includes pathways in which receptor activation stimulates G protein signaling by coupling to the G protein α subunits such as G_αi_, G_αq_, and G_α12/13_ [9]. In one of the canonical signaling pathways, activation of Gαq leads to it coupling to phospholipase C (PLC), thereby activating the PLC-mediated hydrolysis of phosphatidylinositol 4,5-biphosphate (PIP_2_) to diacylglycerol (DAG) and inositol 1,4,5-triphosphate (IP_3_). IP_3_ then initiates Ca^2+^ release into the cytosol [9]. Upon elevated cytosolic Ca^2+^ concentration, proline-rich protein tyrosine kinase 2 becomes activated through phosphorylation at the Y402 residue [10,11]. This can further activate p85 through direct interaction, leading to the initiation of the PI3-kinase (PI3K)/Akt signaling pathway [9,12]. It is generally accepted that Akt activates NF-κB by phosphorylating the Ser 536 residue during skin photoaging [13]. However, the downstream signaling pathways of the PAR2-mediated signaling pathway in the induction of the inflammatory response during skin photoaging is not clearly defined.

Among the downstream mediators of PI3K/Akt, forkhead box O (FoxO) transcription factors are the main downstream mediators of Akt [14]. FoxOs are negatively regulated by Akt signaling and are known to exert inhibitory effects on cell proliferation in various cell types. In skin, PI3K signaling regulates keratinocyte proliferation by activating Akt and its subsequent target molecules, including FoxO. For example, in psoriasis, an immune-mediated inflammatory disease, activation of PI3K/Akt and loss of FoxOs have been observed [15]. In the search for regulatory transcription factors that have activity during oxidative stress, FoxO6 has been recently reported for its protective role by inducing antioxidant gene expression during intrinsic and extrinsic skin aging [16]. This study demonstrated that treating UVB-irradiated B16F10 cells with FoxO6 suppressed intracellular ROS and peroxynitrite (ONOO^−^) levels, subsequently leading to decreased melanin content. Such suppressive effects on the melanin content were not observed in the FoxO6 knockdown experiment [16]. In addition to skin, FoxO6 transactivates the antioxidant genes MnSOD and catalase in human liver cancer cells [17]. As elevated oxidative stress is one of the major characteristics of skin photoaging, the modulation of intracellular antioxidant enzymes through FoxO6 could play an essential role in ameliorating accelerated aging.

In this study, we investigated whether PAR2-mediated Akt activation could phosphorylate NF-κB and FoxO6 and induce cytokines and suppress antioxidative enzymes MnSOD and catalase, which could subsequently promote the intracellular inflammatory response and ROS production, respectively. Our data showed that the PAR2-mediated Akt/NF-κB/FoxO6 signaling pathway led to ROS-mediated inflammation during skin photoaging, suggesting that this signaling axis can be an efficient therapeutic target for the prevention of skin photoaging.

## 2 Method and materials

### 2.1 Reagents

Rabbit polyclonal antibodies to IL-1β, catalase, and p-Akt (Ser473) were purchased from Santa Cruz Biotechnology (Santa Cruz, CA, USA); mouse monoclonal antibodies to α-tubulin, PAR2, Gαq, p65, and TFIIB were purchased from Santa Cruz Biotechnology; goat polyclonal antibody to MnSOD was purchased from Santa Cruz Biotechnology; rabbit polyclonal antibodies to IL-6, tissue factor, and p-p65 (S536) were purchased from Abcam (Cambridge, UK); rabbit monoclonal antibodies to PI3K (p85) and Akt were purchased from Cell Signaling Technology (Danvers, MA, USA); rabbit polyclonal antibodies to FoxO6 and p-FoxO6 (S184) were kindly provided by Dr. H. Henry Dong (University of Pittsburgh, PA, USA); horseradish peroxidase-conjugated anti-rabbit IgG, anti-mouse IgG, and anti-goat IgG were purchased from GeneTex (Irvine, CA, USA). PAR2-small interfering RNA (siRNA) was purchased from Santa Cruz Biotechnology. H_2_DCFDA was purchased from Molecular Probes (Eugene, OR, USA). GB83 was purchased from Axon MedChem (Groningen, Netherlands). SLIGRL-NH_2_ was purchased from Cayman Chemical (Ann Arbor, MI, USA). LY294002 was purchased from Cell Signaling Technology. Lipofectamine 3000 was purchased from Thermo Fisher Scientific (Carlsbad, CA, USA). Dulbecco's phosphate-buffered saline (PBS) was purchased from Gibco (Carlsbad, CA, USA). Dulbecco's modified Eagle's medium was purchased from Welgene (Gyeongsan, South Korea). RiboEx was purchased from GeneAll (Seoul, South Korea). Broad-range protein molecular weight markers were purchased from Elpis-Biotech (Daejeon, South Korea). An adenoviral vector expressing the constitutively active form of FoxO6 was kindly provided by Dr. H. Henry Dong (University of Pittsburgh, PA, USA), as described previously [18].

### 2.2 Cells

The human keratinocyte cell line (HaCaT) was obtained from the American Type Culture Collection (Manassas, VA, USA). Cells were cultured in Dulbecco's modified Eagle's medium (Welgene) containing 4500 mg/L d-glucose, l-glutamine, 110 mg/L sodium pyruvate, sodium bicarbonate, 100 g/mL streptomycin, and 10% heat-inactivated fetal bovine serum. Cells were maintained at 37 °C in 5% CO_2_.

### 2.3 Mice

HRM-2 hairless mice (8 weeks old, male) were purchased from Hoshino Laboratory Animals (Saitama, Japan). The mice were housed under a 12 h/12 h light/dark cycle and given ad libitum access to standard laboratory diet and water. Mice were exposed to UVB radiation (UVP CL-1000) at 150 mJ/cm^2^ every other day for 28 days to induce skin photoaging. After 28 days of the experiment, mice were euthanized with carbon-dioxide and the dorsal skin tissue was obtained and quickly frozen in liquid nitrogen for additional analysis. For histological analysis, the obtained skin was fixed in 10% formalin. In additional experiment, hairless mice were topically applied with the PAR2-specific antagonist GB83. GB83 was dissolved in a vehicle mixed with ethanol and propylene glycol at a ratio of 3:7 and then applied daily at 0.4 μM or 5 μM to the dorsal surface of the mice skin. The mice were exposed to UVB (UVP CL-1000) at 150 mJ/cm^2^ every other day for 28 days. After 28 days, dorsal skin was obtained and frozen in a liquid nitrogen tank for additional analysis. These animal experiments were approved by the Pusan National University Institutional Animal Care and Use Committee (Approval Number PNU-2020-2733).

Homozygous PAR2-knockout (KO) mice of the PAR2−/−; strain and 8-week-old male B6.Cg-*F2RL1*^tm1Mslb^/J mice were kindly provided by Dr. Hak-Sun Yu (Department of Parasitology and Tropical Medicine, School of Medicine, Pusan National University, South Korea). After a 1-week habituation period, mice were exposed to UVB radiation (UVP CL-1000) at 90 mJ/cm^2^ every other day for 3 weeks. Due to greater acute responses observed in the strain, mice were exposed to lower UVB dosage than that of HRM2 hairless mice. After 3 weeks of the experiment, mice were euthanized with carbon-dioxide and the dorsal skin was obtained and quickly frozen in liquid nitrogen for additional analysis. This experiment was reviewed and approved by the Pusan National University Institutional Animal Care and Use Committee (Approval Number PNU-2020-2615).

### 2.4 Cell transfection

Cell transfection was performed using Lipofectamine 3000 (Invitrogen, Carlsbad, CA, USA). Briefly, 6 × 10^5^ cells per well were seeded in 6-well plates and incubated at 37 °C in humidified 5% CO_2_ atmosphere. When the seeded cells reached approximately 70% confluence, they were incubated with PAR2 plasmid (2 μg) and Lipofectamine 3000 complex in normal growth media for 24 h at 37 °C in humidified 5% CO_2_ atmosphere. Then, cells were washed with ice-cold 1X PBS and pellets were collected at 12,000 x *g* at 4 °C for 15 min for further analysis. The pellets were resuspended in total lysis buffer solution composed of NaCl (150 mM), Triton X-100 (1%), sodium deoxycholate (1%), SDS (0.1%), Tris-HCl pH 7.5 (50 mM), EDTA (2 mM, pH 8.0) supplemented with protease inhibitor and phosphatase inhibitors for extraction of total protein from the cells. The human PAR2 construct for cell transfection was kindly provided by Dr. Morley Hollenberg (University of Calgary, Calgary, CA).

### 2.5 siRNA-mediated gene silencing

Pre-designed PAR2 siRNA was purchased from Santa Cruz Biotechnology. siRNA was transfected using Lipofectamine 3000 (Invitrogen) following the manufacturer's protocol. Cells were seeded to be approximately 50–60% confluent at the time of transfection. The final concentration of siRNA was 10 nM. Cells were incubated at 37 °C in 5% CO_2_ for 24 h prior to transfection.

### 2.6 Separations of cytosol and nuclear extraction in skin tissue

Frozen skin tissues (150–200 mg) were ground using liquid nitrogen in a mortar and pestle. Ground skin tissues were homogenized in 1 mL hypotonic lysis buffer. Buffer A was composed of KCl (10 mM), MgCl_2_ (2 mM), dithiothreitol (DTT) (1 mM), EDTA (0.1 mM), PMSF (0.1 mM), pepstatin (1 μM), leupeptin (2 μM), β-glycerophosphate (20 mM), NaF (20 mM), Na_3_VO_4_ (2 mM), and 4-(2-hydroxyethyl)-1-piperazineethanesulfonic acid (HEPES), pH 7.4 (10 mM). The tissue homogenizer was used for 20 s. After homogenates were incubated on ice for 15 min, 125 μL of 10% Nonidet P-40 (NP-40) was added to homogenates and mixed for 15 s, and the mixture was centrifuged at 14,000 *× g* for 2 min at 4 °C. The supernatants were obtained as the cytosol. The pelleted nucleus fraction was washed with 400 μL of buffer A plus 25 μL of 10% NP-40, centrifuged, incubated in 200 μL buffer C [KCl (50 mM), NaCl (300 mM), PMSF (0.1 mM), 10% (v/v) glycerol, pepstatin (1 μM), leupeptin (2 μM), β-glycerophosphate (20 mM), NaF (20 mM), Na_3_VO_4_ (2 mM), HEPES pH 7.8, (50 mM)], incubated in ice for 30 min, and centrifuged at 14,000×*g* for 10 min at 4 °C. The supernatant (nucleus fraction) was harvested and stored at −80 °C for long-term storage.

### 2.7 Immunoprecipitation

Cytosol fractions were immunoprecipitated in immunoprecipitation (IP) buffer composed of Tris-HCl (pH 7.6) (40 mM), NaCl (120 mM), β-glycerophosphate (20 mM), NaF (20 mM), Na_3_VO_4_ (2 mM), PMSF (1 mM), EDTA (5 mM), NP-40 (0.1%), leupeptin (2 μg/mL), aprotinin (1 μg/mL), and pepstatin A (1 μg/mL). Aliquots of cytosol extracts were then pre-cleared using 50% (v/v) protein G agarose for 30 min at 4 °C, centrifuged at 12,000×*g* at 4 °C for 15 min, incubated at 4 °C overnight with the appropriate antibody, and then further incubated overnight at 4 °C with a 50% protein G agarose slurry. After washing the immunoprecipitates three times with IP buffer, immunoprecipitated proteins were detected using sodium dodecyl sulfate (SDS)-PAGE and western blotting was performed.

### 2.8 Histological analysis of skin tissue

Skins were fixed in 10% formalin, embedded in paraffin and 5 μm sections were stained with hematoxylin and eosin (H&E) and was examined using Motic AE31 Inverted Microscope (Motic, Kowloon Bay, Hong Kong).

### 2.9 Measurement of ROS production

ROS production was measured using 2′,7′-dichlorofluorescein diacetate (DCFDA) protocol. Briefly, nonfluorescent DCFDA was oxidized to the highly fluorescent 2,7′-dichlorofluorescein (DCF) in the presence of intracellular esterase and reactive species. DCFDA (25 μM) was added to the skin cytosol fraction to obtain a total volume of 250 μL. Fluorescence intensity was measured and quantified every 5 min for a total of 30 min using a fluorescence plate reader at an excitation wavelength of 485 nm and emission wavelength of 535 nm.

### 2.10 Immunohistochemistry

For immunostaining, skin sections were treated with 3% H_2_O_2_ in distilled water to block residual peroxidase for 10 min at RT. After treatment, skin sections were incubated with Tris-buffered saline (TBS) containing 0.1% Triton-X-100 and normal goat serum at 37 °C for 1 h, and then incubated with PAR2 primary antibody (1:200 dilution) (Santa Cruz Biotechnology) in TBS-T at 4 °C overnight. Sections were then further incubated with secondary goat anti-mouse IgG-horseradish peroxidase-conjugated (HRP) antibody (1:500 dilution) (Santa Cruz Biotechnology) at RT for 1 h. Sections were then stained with diaminobenzidine (DAB) solution, mounted with Dako mounting medium (Dako, Glostrup, Denmark), and covered with cover slips. Stained images were acquired using a Motic AE31 Inverted Microscope (Motic, Kowloon Bay, Hong Kong).

### 2.11 Western blot analysis

Homogenized skin protein samples were boiled for 5 min with 5X SDS loading sample buffer composed of 250 mM Tris-HCl, 10% SDS, 30% glycerol, 5% β-mercapitalethanol, and 0.02% bromophenol blue. The same amount of protein (8–10 μg) was loaded and separated via SDS-PAGE using 7–15% gels and then transferred to PVDF membranes at 25 V for 10 min using a semi-dry transfer method. Membranes were then immediately incubated with a blocking buffer consisting of 10 mM Tris (pH 7.5), 100 mM NaCl, and 0.1% Tween-20 containing 5% non-fat milk. Membranes were blocked for 1 h at RT and then incubated with specific primary antibodies (1:1000–1:2000 dilution) at 4 °C overnight on a shaker. This was followed by HRP-conjugated secondary incubation (1:10000 dilution) for 1 h at RT. Antibody labeling was detected using enhanced chemiluminescence according to the instructions provided by the manufacturer. Molecular weights were determined using broad-range protein markers.

### 2.12 Reverse transcription and real-time quantitative reverse transcription polymerase chain reaction (qRT-PCR)

Frozen skin tissues (150–200 mg) were excised and ground in a mortar and pestle in liquid nitrogen. The ground skin tissues were used for RNA isolation using the RNeasy Mini Kit (Qiagen, Hilden, Germany). Total amount of 2 μg of total RNA was used to synthesize cDNA. qRT-PCR analysis was performed to detect mRNA levels using the SYBR Green and CFX Connect System (Bio-Rad Laboratories Inc., Hercules, CA, USA). All primers were designed and purchased from Bioneer (Daejeon, South Korea). The primer sequences used are listed in Supplementary Tables 1 and 2 The primer concentration used for qRT-PCR analysis was at 10 pmol concentration.

### 2.13 Statistical analyses

Analysis of variance was used to determine the statistical significance of the differences among the groups. Fisher's protected least significant difference post-hoc test was used to test the significant differences between group means. P-values < 0.05 were considered statistically significant.

## 3 Results

### 3.1 UVB-induced skin photoaging was associated with increased oxidative stress and inflammatory responses in hairless mice

**Fig. 1:**
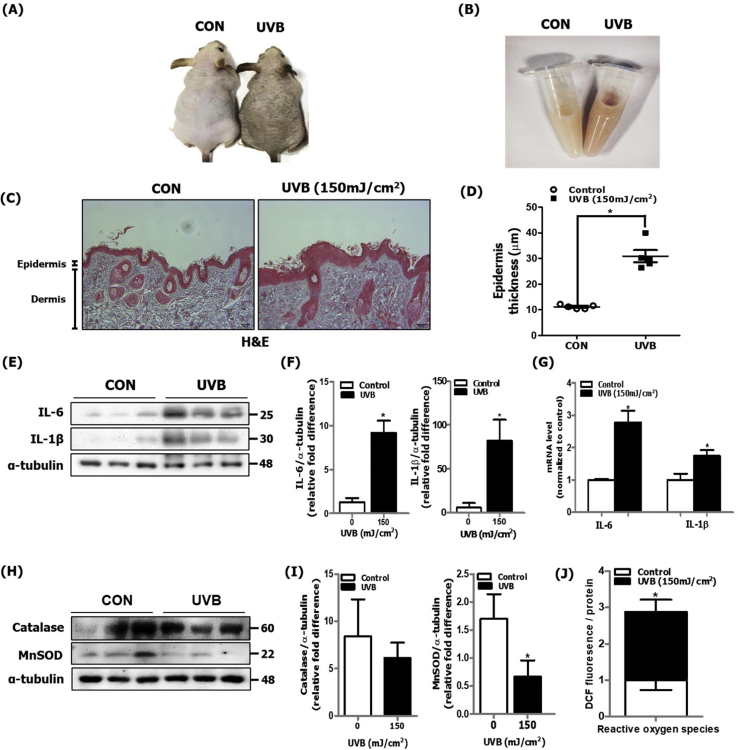
Increased inflammatory response and oxidative stress in UVB-irradiated dorsal skin of hairless mice. After HRM2 mice were repeatedly exposed to UVB radiation (150 mJ/cm^2^) for 28 days, skin tissue was excised and homogenized to separate the nuclear and cytosolic fractions (N = 5 per group). (A) A photograph of the dorsal skin of control and UVB-irradiated mice was taken for phenotype analysis. (B) A photograph of skin tissue homogenates was taken to visualize discoloration. (C) H&E-stained histological image of skin section of control and UVB-irradiated mice was captured and (D) epidermis thickness was quantified using Motic Image Plus 2.0 software (N = 5 per group). (E) The protein expression levels of IL-6 and IL-1β were measured using western blotting and (F) quantified using ImageJ software. α-tubulin was the loading control of the cytosolic fractions. (G) mRNA levels of IL-6 and IL-1β were quantified using qPCR (N = 5 per group). (H) The protein levels of catalase and MnSOD were measured using western blotting and (I) quantified using ImageJ software. α-tubulin was the loading control of the cytosolic fractions. (J) ROS production was determined by measuring the DCF fluorescence level in the skin cytosolic fraction (N = 5 per group). All data are represented as the mean ± SEM, and significance was determined using an unpaired *t*-test; *P < 0.05 vs. control.

In skin exposed to long-term, persistent UV irradiation, ROS production and the inflammatory response is promoted, which is mediated by the activation of diverse intracellular signaling molecules such as NF-κB [19]. We irradiated dorsal skin of hairless mice with UVB (150 mJ/cm^2^) every other day for 28 days. We confirmed the induction of oxidative stress and the inflammatory response in our *in vivo* experimental skin photoaging model. We observed that the lightness of UVB-irradiated dorsal skin of hairless mice was significantly decreased in comparison to control mice skin (Fig. 1A). Consistently, UVB-irradiated dorsal skin homogenates showed discoloration and was darker than that of control mice skin (Fig. 1B), suggesting greater melanin content. To further verify induction of skin photoaging, we examined skin by H&E staining and observed a significant increase in the thickness of the epidermis of UVB-irradiated mice skin in comparison to control mice (Fig. 1C and D). At the molecular level, we measured proinflammatory cytokines and observed a notable increase in both protein and mRNA expression levels of IL-6 and IL-1β (Fig. 1E, F and G). The antioxidative enzyme MnSOD was observed to be downregulated in UVB-irradiated dorsal skin compared to that in control mice skin, whereas no changed was observed in catalase level (Fig. 1H and I). As a consequence of decreased levels of antioxidant enzyme, we observed increased ROS production in the skin cytosolic fraction (Fig. 1J). These data indicate that characteristics of UVB-induced skin photoaging were observed at both phenotypical and molecular levels in hairless mice.

### 3.2 UVB-induced skin photoaging was associated with increased PAR2/Akt pathway and NF-κB/FoxO6 modulation in hairless mice

**Fig. 2:**
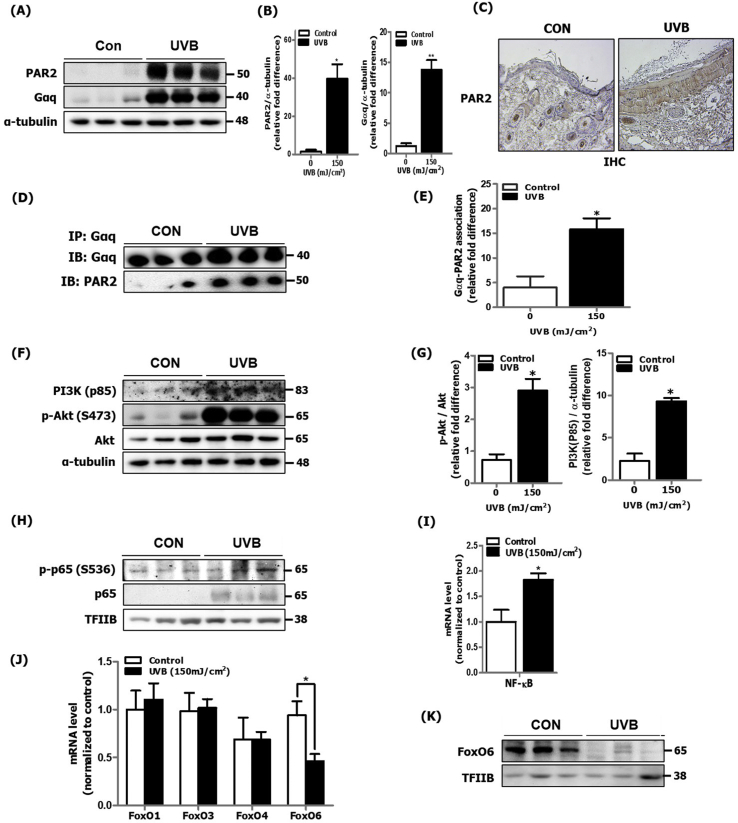
Increased PAR2 expression and Akt/NF-κB/FoxO6 modulation in UVB-irradiated dorsal skin of hairless mice. UVB-irradiated mice skin was homogenized for mRNA and protein extraction (N = 5 per group). (A) The protein expression level of PAR2 and the Gαq subunit was detected in cytosol using western blotting and (B) quantified using ImageJ software (N = 3 per group). α-tubulin was the loading control of the cytosolic fractions. (C) PAR2 localization was examined in skin sections using immunohistochemistry. (D) The level of physical association between PAR2 and Gαq was examined using immunoprecipitation and (E) quantified using ImageJ software (N = 3 per group). (F) PI3K and Akt phosphorylation was detected in the cytosol using western blotting and (G) quantified using ImageJ software (N = 3 per group). α-tubulin was the loading control of the cytosolic fractions. (H) The activation level of p65 was measured by detecting p65 phosphorylation in the nucleus using western blotting and (I) quantified mRNA level using qRT-PCR (N = 5 per group). TFIIB was the loading control of the nuclear fraction. (J) The mRNA expression levels of FoxO isoforms were investigated using qRT-PCR (N = 5 per group). (K) The protein expression level of FoxO6 was detected using western blotting. TFIIB was the loading control of the nuclear fraction. All data are represented as the mean ± SEM and significance was determined using an unpaired *t*-test; *P < 0.05 vs. control.

Previous studies have reported that PAR2 expression is upregulated in UVB-irradiated human skin, suggesting its role in skin inflammation [7]. Here, we confirmed that protein expression of PAR2 and G-protein Gαq subunit was notably upregulated where the PAR2 expression was particularly increased in the epidermis area of skin in UVB-irradiated mice in comparison to that in control mice (Fig. 2A, B and C). In the canonical signaling pathway, Gαq is an upstream G-protein subunit that mediates the release of endoplasmic reticulum (ER)-stored calcium into the cytosol, which is well observed in UVB-irradiated skin. In general, such cellular effects are mediated through activation of the PLC-IP_3_ signaling pathway [20]. To confirm whether UVB-irradiated skin inflammation is mediated by PAR2 coupling to the Gαq subunit, we investigated the physical interaction between PAR2 and Gαq in the UVB-irradiated skin cytosolic fraction. The degree of physical association between PAR2 and Gαq was notably increased in UVB-irradiated mice skin compared to that in control mice skin (Fig. 2D and E). Furthermore, we considered the PLC-mediated PI3K/Akt activation pathway for cell proliferative effects [21] observed during skin photoaging. We observed an increase in PI3K and Akt phosphorylation (Ser473) in UVB-irradiated skin of hairless mice (Fig. 2F and G). As is well established, we confirmed increased p65 phosphorylation by Akt at Ser536 in the nucleus (Fig. 2H) and p65 mRNA level (Fig. 2I). Although not much is known about its role in inflammation during skin photoaging, we detected that mRNA level of FoxO6 was decreased, among other FoxO isoforms (Fig. 2J). And the protein level of FoxO6 was decreased in the nucleus (Fig. 2K). These data indicate that PAR2 upregulation occurs during skin photoaging, suggesting its potential association with the Akt/NF-κB-mediated inflammatory response and Akt/FoxO6-mediated MnSOD suppression. This upregulation leads to increased ROS production, further exacerbating the inflammatory response during skin photoaging.

### 3.3 PAR2 inhibition suppressed oxidative stress and inflammatory response in hairless mice during skin photoaging

**Fig. 3:**
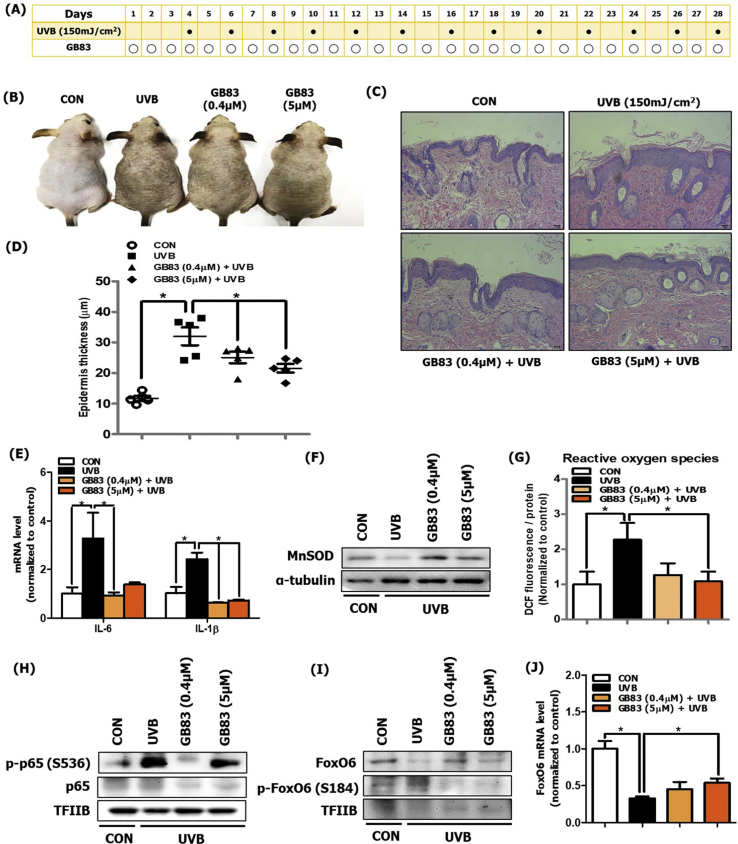
PAR2 inhibition decreased oxidative stress and inflammation in UVB-irradiated dorsal skin of hairless mice. (A) Approved experimental procedure for topical application of GB83 during skin photoaging (N = 5 per group). (B) A photograph of the dorsal skin of control, UVB-treated, and GB83 with UVB-treated hairless mice was taken for phenotype analysis. (C) H&E-stained histological image of a dorsal skin section from the skin of control, UVB-treated, and GB83 with UVB-treated hairless mice was captured. (D) The thickness of the epidermis was quantified using Motic Image Plus 2.0 software (N = 5 per group). (E) The mRNA expression levels of IL-6 and IL-1β were measured using qRT-PCR (N = 5 per group). (F) The protein expression level of MnSOD was measured using western blotting (N = 3 per group). α-tubulin was the loading control of the cytosolic fractions. (G) The ROS production level was measured using the DCF fluorescence level in skin cytosol fraction (N = 5 per group). (H) The protein expression levels of p-p65 and p65 and (I) p-FoxO6 and FoxO6 were measured using western blotting (N = 3 per group). TFIIB was the loading control of the nuclear fraction. (J) The mRNA expression level of FoxO6 was measured using qRT-PCR (N = 5 per group). All data are represented as the mean ± SEM and significance was determined using an one-factor analysis of variance (ANOVA); *P < 0.05.

To determine the role of PAR2 in oxidation and inflammatory responses through Akt/NF-κB/FoxO6 modulation, we used the PAR2-specific antagonist, GB83. GB83 was dorsally applied to the UVB-irradiated skin of hairless mice (Fig. 3A). The PAR2 antagonist significantly suppressed both UVB-induced skin discoloration and epidermal thickness in comparison to control mice skin (Fig. 3B, C and D). We next measured the mRNA levels of IL-6 and IL-1β, which were decreased by GB83 at both low and high doses (Fig. 3E). We also measured the expression of MnSOD, which was upregulated by GB83 treatment at both low and high doses in comparison to in vehicle-treated UVB-irradiated mice skin (Fig. 3F). Subsequently, we measured ROS production with DCF fluorescence and found that ROS levels were decreased by GB83 treatment at both low and high doses in comparison to vehicle-treated UVB-irradiated mice skin (Fig. 3G). At the molecular level, we detected that p65 phosphorylation in the nucleus was suppressed by GB83 treatment, notably at low doses, in comparison to that in vehicle-treated UVB-irradiated mice skin (Fig. 3H). The protein and mRNA expression of FoxO6 was increased by GB83 treatment at both low and high doses in comparison to vehicle-treated UVB-irradiated mice skin. Otherwise, FoxO6 phosphorylation (inactivated form) in the nucleus was suppressed by GB83 treatment (Fig. 3I and J). These results confirm that PAR2 mediates oxidative stress and inflammation during skin photoaging.

### 3.4 PAR2 KO mice exhibited decreased oxidative stress and inflammation during skin photoaging

**Fig. 4:**
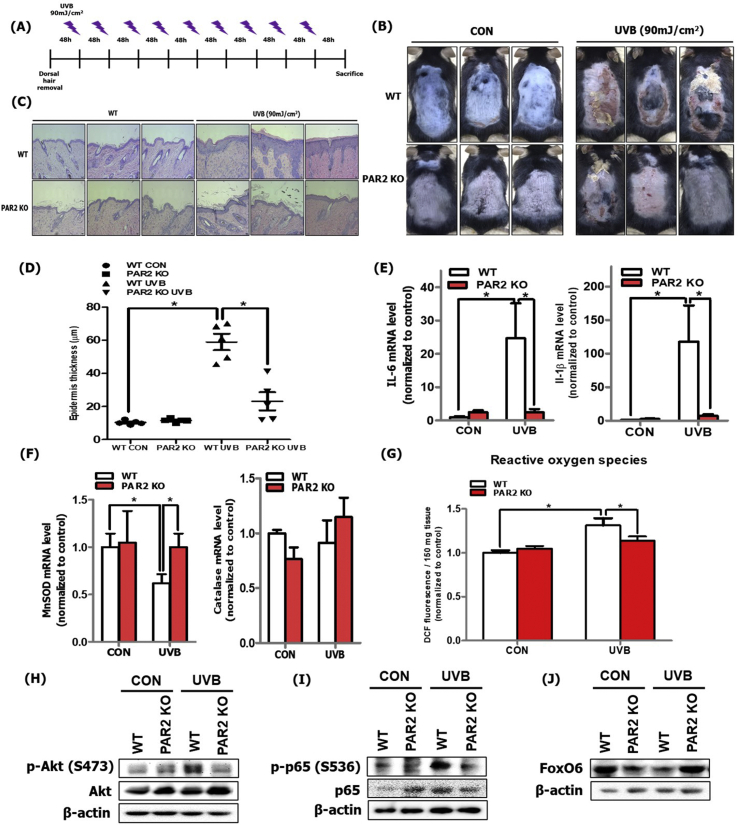
PAR2 KO mice showed reduced oxidative stress and inflammation during skin photoaging. (A) The experimental procedure for induction of UVB-irradiated skin photoaging using C57BL/6-strained WT and PAR2 KO mice (N = 5 per group). (B) Photographs of dorsal skin of control and UVB-irradiated mice of WT and PAR2 KO mice were taken for phenotype analysis. (B) H&E-stained histological image of a skin section of the control and UVB-irradiated WT and PAR2 KO mice was captured and (C) epidermal thickness was measured and (D) quantified using Motic Image Plus 2.0 software (N = 5 per group). (E) The mRNA levels of IL-6 and IL-1β and (F) MnSOD and catalase were quantified using qRT-PCR (N = 5 per group). (G) ROS production was determined by measuring DCF fluorescence level in the skin cytosol fraction (N = 5 per group). (H) The protein expression levels of phosphorylated Akt, total-Akt (I) phosphorylated p65, p65, and (J) total FoxO6 were detected using western blotting (N = 5 per group). β-actin was the loading control. All data are represented as the mean ± SEM, and significance was determined using an one-factor ANOVA; *P < 0.05.

To confirm the pivotal role of PAR2 in oxidative stress and inflammatory responses, we used PAR2-deficient (PAR2 KO) mice. The mice were subjected to UVB irradiation (90 mJ/cm^2^) every other day for 3 weeks (Fig. 4A). We first compared the dorsal skin photographs of wild-type (WT) and PAR2 KO mice with or without UVB irradiation at the end of the experiment. The UVB-irradiated PAR2 KO mice showed suppressed skin barrier destruction and epidermal thickness in comparison to UVB-irradiated WT mice (Fig. 4B, C and D). To investigate changes in the inflammatory response, we measured the mRNA levels of IL-6 and IL-1β and demonstrated that these were suppressed in UVB-irradiated PAR2 KO mice in comparison to those in UVB-irradiated WT mice (Fig. 4E). We detected mRNA levels of MnSOD and catalase and found that MnSOD was increased in UVB-irradiated PAR2 KO mice compared to that in UVB-irradiated WT mice. Otherwise, catalase mRNA level was not significantly changed (Fig. 4F). Similarly, the level of ROS production was suppressed in UVB-irradiated PAR2 KO mice in comparison to in UVB-irradiated WT mice (Fig. 4G). The phosphorylation levels of Akt and p65 were also suppressed in UVB-irradiated PAR2 KO mice in comparison to that in UVB-irradiated WT mice (Fig. 4H and I). FoxO6 levels were increased in UVB-irradiated PAR2 KO mice compared to that in UVB-irradiated WT mice (Fig. 4J). These results confirm the PAR2-specific regulatory role in oxidative stress and inflammation through Akt/NF-κB/FoxO6 signaling modulation during skin photoaging.

### 3.5 PAR2 induced inflammatory response through the Akt/NF-kB/FoxO6 signaling pathway in HaCaT cells

**Fig. 5:**
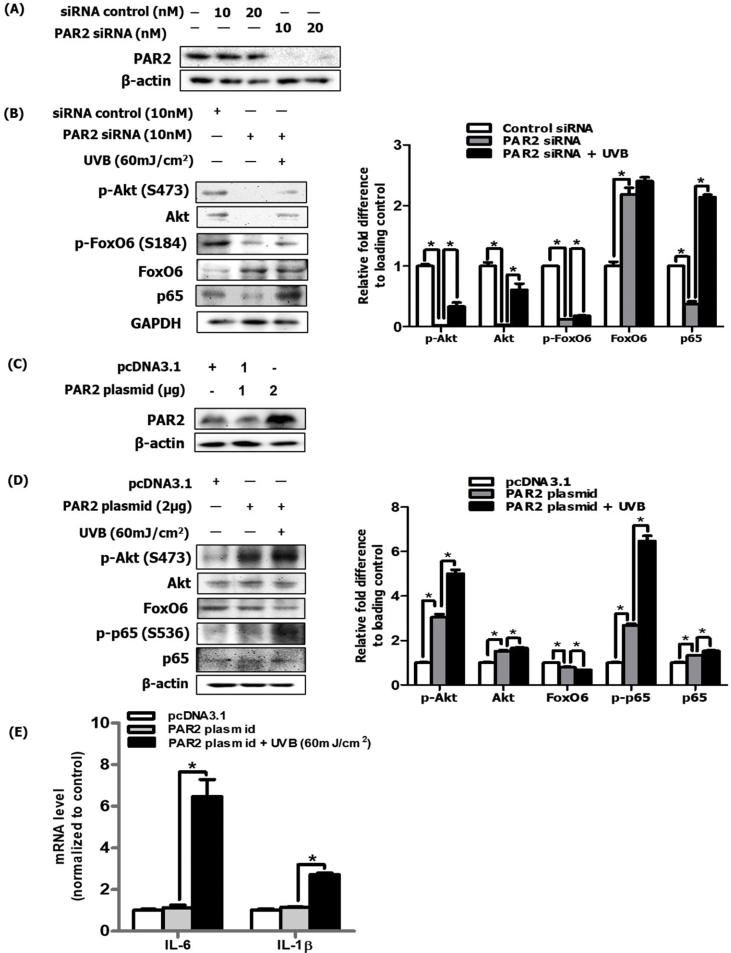
PAR2 induced oxidative stress and the inflammatory response through the Akt/NF-κB/FoxO6 signaling pathway in HaCaT cells. (A) After PAR2 knockdown using siRNA, PAR2 protein level was analyzed via western blotting and observed PAR2 was efficiently knocked-down at 10 nM. β-actin was the loading control of the cytosolic fractions. (B) After treatment with 10 nM siRNA under UVB-irradiated condition, cell lysates were analyzed via western blotting for phosphorylated Akt, total-Akt, phosphorylated FoxO6, total-FoxO6, and p65 and were quantified using ImageJ software (N = 3). GAPDH was the loading control of the whole lysis. (C) The protein expression levels of PAR2 were measured in the HaCaT cell line expressing pcDNA3, a PAR2 plasmid, using transfection method. β-actin was the loading control of the cytosolic fractions. (D) The protein expression levels for phosphorylated Akt, total-Akt, FoxO6, phosphorylated p65 and p65 were measured in the UVB-irradiated HaCaT cells overexpressing PAR2 plasmid and were quantified using ImageJ software (N = 3). β-actin was the loading control of the whole lysis. The mRNA levels of (E) IL-6 and IL-1β were quantified under the same experimental condition. All data are represented as the mean ± SEM (N = 3 per group) and significance was determined using an one-factor ANOVA; *P < 0.05.

To confirm the *in vivo* results, we performed an *in vitro* experiment using UVB-irradiated HaCaT cells. We deleted PAR2 using si-PAR2, which was efficiently achieved at a concentration of 10 nM (Fig. 5A). We treated cells with 10 nM si-PAR2 and observed that Akt and p65 phosphorylation was decreased and FoxO6 was increased (Fig. 5B). To confirm this result, the PAR2 plasmid was transfected in HaCaT cells (Fig. 5C), which led to increase in Akt and p65 phosphorylation and decrease in FoxO6 expression in these cells (Fig. 5D). Next, we examined mRNA levels of IL-6 and IL-1β and demonstrated that they had significantly increased in UVB-treated and PAR2 plasmid-transfected cells (Fig. 5E). These results further confirm the *in vivo* results of PAR2-mediated inflammatory response through modulation of the Akt/NF-κB/FoxO6 signaling pathway.

### 3.6 FoxO6 suppressed PAR2-Akt-mediated ROS production in UVB-irradiated HaCaT cells

**Fig. 6:**
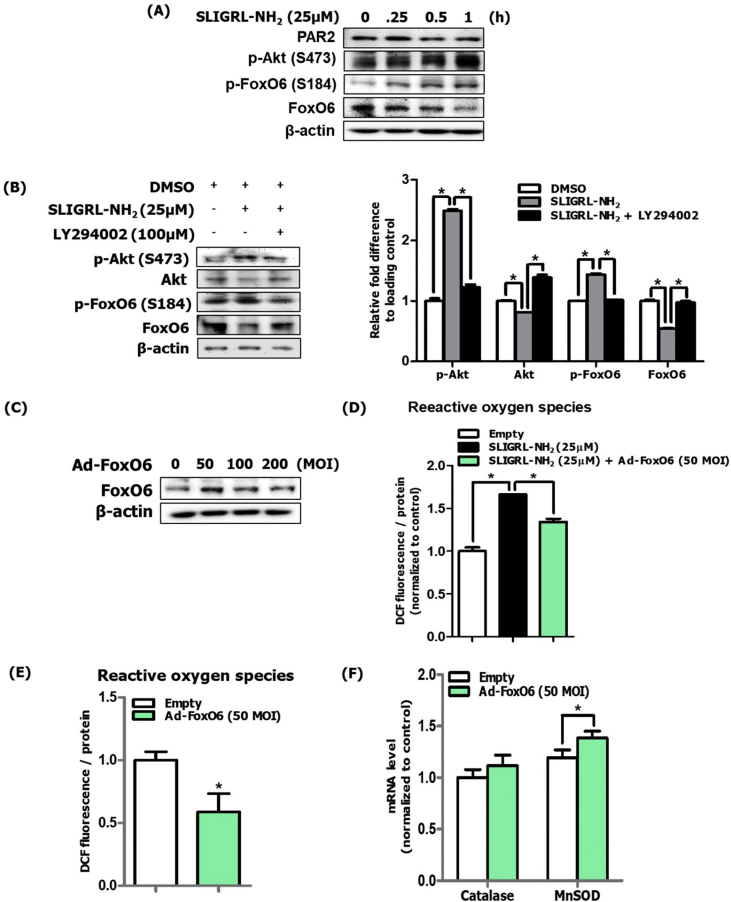

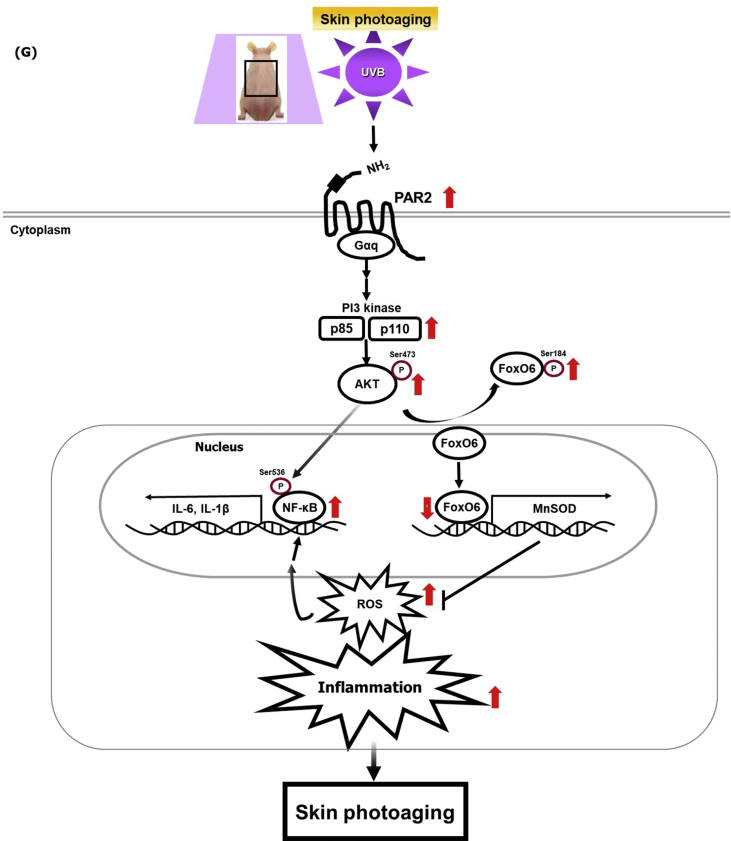
FoxO6 suppressed PAR2-induced ROS production in HaCaT cells. (A) Protein levels of PAR2, p-Akt, p-FoxO6, and FoxO6 in SLIGRL-NH_2_ treated cells. β-actin was the loading control of the whole lysis. (B) Suppression of these phosphorylated Akt and FoxO6 proteins was observed in LY294002-pretreated cells and these were quantified using ImageJ software (N = 3). (C) HaCaT cells were treated with FoxO6 adenovirus dose-dependently, and 50 MOI was the effective treatment dose. β-actin was the loading control of the whole lysis. (D) Suppression of PAR2-induced ROS levels by FoxO6 adenovirus (50 MOI) (E) Suppression of ROS levels by FoxO6 adenovirus (50 MOI). ROS levels in HaCaT cells were measured using DCF fluorescence levels (F) mRNA levels of antioxidant catalase and MnSOD genes were quantified. All data are represented as the mean ± SEM (N = 3 per group) and significance was determined using an unpaired *t*-test and one-factor ANOVA; *P < 0.05. (G) Graphical description of PAR2 inducing ROS-mediated inflammation through Akt-mediated NF-κB and FoxO6 modulation during skin photoaging.

During photoaging, the biological role of FoxO6 was found to play a role in melanogenesis in the B16F10 murine melanoma cell line [16]. However, the role of FoxO6 in inflammation in keratinocytes has not been investigated. To investigate the signaling pathway of Akt-mediated FoxO6 regulation, we used HaCaT cells and treated cells with the PAR2 agonist, SLIGRL-NH_2_. Furthermore, we demonstrated that phosphorylation of Akt and FoxO6 was increased in a timely manner (Fig. 6A), confirming that PAR2 regulates the Akt-FoxO6 axis. Pretreatment with LY294002, a PI3K inhibitor, followed by a post-treatment with a PAR2 agonist suppressed FoxO6 phosphorylation (Fig. 6B). To further investigate the regulatory role of PAR2-mediated FoxO6 in oxidative stress in keratinocytes, we used the constitutively active form of FoxO6 adenovirus for treatment (Fig. 6C). FoxO6 suppressed PAR2 agonist-indued ROS production (Fig. 6D). Furthermore, FoxO6 by its own expression suppressed intracellular ROS levels (Fig. 6E). When we measured the mRNA levels of catalase and MnSOD and observed that Ad-FoxO6 treatment upregulated the mRNA levels of MnSOD gene (Fig. 6F). these results suggest a beneficial role of FoxO6 in suppressing ROS production in keratinocytes. Furthermore, these data suggest a novel role of FoxO6 in suppressing ROS levels, thus decreasing the inflammatory response in keratinocytes.

## 4 Discussion

Acute and chronic exposure to UVB irradiation induces characteristic molecular changes that include ROS formation, DNA and protein damage, and inflammation. These changes cumulatively lead to accelerated skin aging and development of skin cancer [22,23]. Focusing on the inflammatory response, initial biological changes to the skin as a result of UV irradiation are skin redness or erythema due to increased blood vessel dilation and increased vascularization [24,25]. In inflamed skin, angiogenesis and vascular remodeling are characterized by high vasculature permeability, elevated blood flow, inflammatory cell infiltration, and activated vascular endothelial cells expressing cytokines, further exacerbating inflammatory conditions. As such, diverse signaling pathways and action mechanisms have been reported. Here, we show that PAR2 is a critical upstream mediator in both skin inflammation and intracellular oxidative stress during photoaging (Fig. 4E, F and G), based on a phenotype analysis of PAR2-deficient mice. Moreover, our results delineate that Akt-mediated NF-κB and FoxO6 modification is a downstream pathway for the PAR2-induced upregulation of proinflammatory cytokines and downregulation of antioxidative gene transcription in epidermal keratinocytes (Figs. 5 and 6).

PAR2 belongs to a superfamily of GPCRs and can be activated by endogenous and exogenous serine protease enzymes that cleave the extracellular N-terminal domain of the receptor, leaving tethered ligand peptide that acts as an activator of the receptor [26]. Using serine proteases or PAR-specific synthetic peptides, the effects of PAR activation in diverse disease progression were demonstrated in both *in vitro* and *in vivo* experimental models. Previous findings demonstrated the biological roles of PAR2 in the function of innate and immune responses and development of inflammatory and allergic responses [27]. Focusing on the inflammatory response during skin photoaging, it has been determined that the immune system, inflammation, and coagulation are simultaneously activated to defend against potentially damaging stimuli in the skin [28,29].

In the canonical signaling pathway, PAR2 couples with PLC to hydrolyze PIP_2_ into DAG and IP_3_, which binds with IP_3_R on the ER membrane and subsequently releases ER calcium into the cytosol, whereas DAG activates protein kinase C (PKC) [30,31]. In keratinocytes, the PLC signaling pathway been demonstrated to play a critical regulatory role in skin inflammation using a PLC-deficient mouse model [32,33]. To elucidate the potential downstream signaling pathway of PAR2-mediated PLC activation during photoaging, the role of PI3K-mediated Akt and p65 activation was examined. Akt and p65 activation was observed in the proinflammatory status of mast cells of cutaneous neurofibroma [34]. PI3K/Akt, a well-known downstream signaling kinase of PLC, has been reported to induce cell proliferation and survival. An increase in Akt and NF-κB activation was also reported in both chronologically and extrinsically aged skin with an inflammatory response, whereas *in vivo* inhibition of Akt activity led to suppression of NF-κB activation [13,35]. Although the role of Akt and NF-κB signaling has been reported, its involvement in the PAR2 signaling cascade during skin photoaging has not been defined. Our results in photoaged skin revealed that interaction of PAR2 with Gαq correlated with an increase in PI3K/Akt activation and NF-κB phosphorylation levels (Fig. 2F and H), whereas the reversal effects were observed in PAR2 KO mice (Fig. 4H and I). These reversal effects were confirmed in keratinocytes treated with PAR2 siRNA and irradiated with UVB (Fig. 5B). During UVB irradiation, active metabolism of arachidonic acid to prostaglandin appears to be mediated by the upregulation of COX-2, a rate-limiting enzyme that mediates its metabolic conversion [[36], [37], [38]]. To further emphasize the role of COX-2 during accelerated skin aging, it has been demonstrated to play a critical role in the aging process [39]. During inflammation-associated aging progression, the expressions of cytokines such as IL-6 and IL-1β and COX-2 mRNA and protein are upregulated by redox-sensitive transcription factor NF-κB; these molecules act as a free radical or ROS source, leading to increased oxidative stress during aging [40]. Based on our data, the PAR2-Akt axis mediates NF-κB activation, which induces upregulation of cytokines as well as COX-2 (data not shown). These findings emphasize the regulatory role of PAR2-mediated Akt activation in the ROS production and inflammatory response during skin photoaging.

Inhibition of PAR2 activation using GB83 decreased the inflammatory response and oxidative stress and decreased NF-κB/FoxO6 phosphorylation during skin photoaging (Fig. 3). Furthermore, PAR2 deficiency in the skin is known for its anti-inflammatory effects. Using photoaged PAR2 KO mice, we demonstrated that such NF-κB phosphorylation and FoxO6 suppression were reversed (Fig. 4I and J). In keratinocytes, PAR2 siRNA treatment with UVB irradiation confirmed these *in vivo* results, whereas PAR2 plasmid overexpression in HaCaT cells irradiated with UVB reversed these effects (Fig. 5). Treatment of adenoviral FoxO6 suppressed PAR2-mediated ROS production (Fig. 6D), emphasizing its protective role against ROS and inflammation in keratinocytes. These data further support the previous report that showed suppression of the inflammatory response and itching in atopic dermatitis by treatment with pepducin, a PAR2 signaling inhibitor [41]. Another study demonstrated the delayed onset of inflammation with defects in P-selectin-mediated leukocyte rolling in PAR2-deficient mice in comparison to WT mice [42]. PI3K/Akt-mediated FoxO6 has been reported to have a redox regulatory role by increasing antioxidative gene expression levels and inhibition of proinflammatory mediators [17,[43], [44], [45], [46]]. Akt deficiency led to resistance to H_2_O_2_, which caused premature senescence in mouse embryonic fibroblasts (MEFs); this effect was abrogated by overexpression of loss-of-function FoxO in MEFs [46]. Transcriptionally inactive p-FoxO6 is unable to induce its target antioxidative functional enzymes, MnSOD and catalase, thus failing to protect from ROS production [47]. In photoaged skin, Akt activation suppresses transcriptionally active FoxO6 levels, which subsequently leads to an enhancement of oxidative stress in the skin. In turn, this process leads to microphthalmia-associated transcription factor (MITF)-mediated skin melanogenesis in UVB-irradiated murine melanoma cells [16]. To further elucidate the potential relationship between FoxO6 and proinflammatory mediators, it was previously shown that FoxO6 interacts with NF-κB in endotoxin-induced inflammation through Akt phosphorylation in the liver [17]. For the first time, we examined and demonstrated the upstream regulatory role of PAR2 for the antioxidative transcription factor FoxO6 in the oxidative response during skin photoaging *in vivo* (Fig. 3, Fig. 4J). In the keratinocyte cell line, FoxO6 overexpression using adenoviral FoxO6 upregulated MnSOD and led to suppression of ROS (Fig. 6E and F). The current study is limited by the detailed mode of FoxO6 activity, including posttranslational modifications, subcellular localization, interaction with coregulators, and stability [47]. Therefore, further studies are necessary to examine PI3K/Akt-mediated FoxO6 modification during skin photoaging. It is of interest to investigate whether FoxO6 could directly interact with proinflammatory mediators to suppress its inflammatory effects as a defense mechanism during skin photoaging.

In summary, for the first time, we showed that PAR2-Gαq mediated the elevation of ROS and inflammation in PI3K/Akt-mediated phosphorylation of NF-κB (S536) in a FoxO6 (S184)-dependent manner, both *in vivo* and *in vitro*. Our data demonstrated that PAR2-Gαq couples and induces oxidative stress and the inflammatory response through Akt/NF-κB/FoxO6 phosphorylation, leading to a subsequent increase in proinflammatory cytokine production and a decrease in antioxidative MnSOD enzyme during skin photoaging (Fig. 6G). The significance of the current finding is that the PAR2-Akt signaling axis is a critical upstream regulator of NF-κB and FoxO6 phosphorylation in the ROS-mediated inflammatory response during skin photoaging. The PAR2-Akt signaling axis could therefore be a potential therapeutic target for managing inflammation in skin photoaging.

## Funding

This work was supported by a National Research Foundation of Korea (NRF) grant funded by the Korean government (MSIT-2018R1A2A3075425) and Global Ph.D. fellowship (2019H1A2A1077276).

## Author contributions

EJ Bang conducted the experiments and wrote the manuscript. D. H. Kim designed the studies and critically reviewed the manuscript. H.Y. Chung supervised, designed, and discussed an overview of the studies and provided comments on the manuscript.

## Declaration of competing interest

There are no conflicts of interest to declare.
