# Supplementary material for: Protease-activated receptor 2 induces ROS-mediated inflammation through Akt-mediated NF-κB and FoxO6 modulation during skin photoaging
Source: Redox Biol. 2021 May 26;44:102022. doi: 10.1016/j.redox.2021.102022 (PMC8182111; doi:10.1016/j.redox.2021.102022)
Supplement: Multimedia component 1 [file mmc1.docx]

**Supplementary Table 1.** qRT-PCR murine primers

| **Gene** | **Primer sequence** |
| --- | --- |
| **PAR2** | 5’- CACCTGGCAAGAAGGCTAAG-3’  5’- CCCAGGGTTACTGACGCTAA-3’ |
| **IL-6** | 5’- TTGCCTTCTTGGGACTGATG-3’  5’- CCACGATTTCCCAGAGAACA-3’ |
| **IL-1β** | 5’- TCCAGGATGAGGACATGAGCC-3’  5’- GAACGTCACACACCAGCAGG-3’ |
| **FoxO1** | 5’- AAGAGCGTGCCCTACTTCAA-3’  5’- CTCTTGCCCAGACTCCAGAG-3’ |
| **FoxO3** | 5’- AGCCGTGTACTGTGGAGCTT-3’  5’- TCTTGGCGGTATATGGGAAG-3’ |
| **FoxO4** | 5’- CAAGAAGAAGCCGTCTGTCC-3’  5’- CTGACGGTGCTAGCATTTGA-3’ |
| **FoxO6** | 5’- CTGGCAAGAGTTCATGGTGG-3’  5’- GTGCAGCTGCTTCTTCTTGC-3’ |
| **Catalase** | 5’- ACATGGTCTGGGACTTCTGG-3’  5’- CAAGTTTTTGATGCCCTGGT-3’ |
| **MnSOD** | 5’- CCGAGGAGAAGTACCACGAG-3’  5’- GCTTGATAGCCTCCAGCAA-3’ |
| **GAPDH** | 5’- AAGGTCATCCCAGAGCTGAA-3’  5’- CTGCTTCACCACCTTCTTGA-3’ |

**Supplementary Table 2.** qRT-PCR human primers

| **Gene** | **Primer sequence** |
| --- | --- |
| **IL-6** | 5’- GTGTGAAAGCAGCAAAGAG -3’  5’- CTCCAAAAGACCAGTGATG-3’ |
| **IL-1β** | 5’- ACAGGCTGCTCTGGGATTCT -3’  5’- TGAAGCCCTTGCTGTAGTGG-3’ |
| **Catalase** | 5’- GCCTGGGACCCAATTATCTT-3’  5’- GAATCTCCGCACTTCTCCAG-3’ |
| **MnSOD** | 5’- TTGGCCAAGGGAGATGTTAC-3’  5’- AGTCACGTTTGATGGCTTCC-3’ |
| **GAPDH** | 5’- TGGTGAAGACGCCAGTGGA-3’  5’- GCACCGTCAAGGCTGAGAAC-3’ |
